# A systematic approach to detecting transcription factors in response to environmental stresses

**DOI:** 10.1186/1471-2105-8-473

**Published:** 2007-12-08

**Authors:** Li-Hsieh Lin, Hsiao-Ching Lee, Wen-Hsiung Li, Bor-Sen Chen

**Affiliations:** 1 Lab of Systems Biology, Department of Electronical Engineering, National Tsing Hua University, 101, Sec 2, Kuang Fu Hsinchu, 300, Taiwan; 2 Department of Life Science & Institute of Bioinformatics and Structural Biology, National Tsing Hua University, Hsinchu, 300, Taiwan; 3 Department of Ecology and Evolution, University of Chicago, USA; 4 Genomics Research Center, Academia Sinica, Taipei, Taiwan

## Abstract

**Background:**

Eukaryotic cells have developed mechanisms to respond to external environmental or physiological changes (stresses). In order to increase the activities of stress-protection functions in response to an environmental change, the internal cell mechanisms need to induce certain specific gene expression patterns and pathways by changing the expression levels of specific transcription factors (TFs). The conventional methods to find these specific TFs and their interactivities are slow and laborious. In this study, a novel efficient method is proposed to detect the TFs and their interactivities that regulate yeast genes that respond to any specific environment change.

**Results:**

For each gene expressed in a specific environmental condition, a dynamic regulatory model is constructed in which the coefficients of the model represent the transcriptional activities and interactivities of the corresponding TFs. The proposed method requires only microarray data and information of all TFs that bind to the gene but it has superior resolution than the current methods. Our method not only can find stress-specific TFs but also can predict their regulatory strengths and interactivities. Moreover, TFs can be ranked, so that we can identify the major TFs to a stress. Similarly, it can rank the interactions between TFs and identify the major cooperative TF pairs. In addition, the cross-talks and interactivities among different stress-induced pathways are specified by the proposed scheme to gain much insight into protective mechanisms of yeast under different environmental stresses.

**Conclusion:**

In this study, we find significant stress-specific and cell cycle-controlled TFs via constructing a transcriptional dynamic model to regulate the expression profiles of genes under different environmental conditions through microarray data. We have applied this TF activity and interactivity detection method to many stress conditions, including hyper- and hypo- osmotic shock, heat shock, hydrogen peroxide and cell cycle, because the available expression time profiles for these conditions are long enough. Especially, we find significant TFs and cooperative TFs responding to environmental changes. Our method may also be applicable to other stresses if the gene expression profiles have been examined for a sufficiently long time.

## Background

Microarray gene expression data can provide a global view of transcriptional regulation, but new methods of analysis are needed to extract biologically meaningful information. The DNA sequence elements that act as binding sites for transcription factors (TFs) coordinate the expression of genes having one or more such elements in their promoter region [1]. Systematic approaches to identifying the biological functions of TFs are needed to ensure rapid progress from genome sequence data to direct experiments and applications [2-7].

A popular approach to analyzing microarray data at present is to cluster genes based on the similarities of their expression profiles. It has been used to identify *cis*-regulatory elements. The rationale is that co-expressed genes are likely to be co-regulated and, therefore, may share common regulatory elements [8]. In addition, Eisen *et al*. [9] constructed a probabilistic model that uses expression data to link regulators to regulated genes. Their method assumes that the expression levels of regulated genes depend on the expression levels of their regulators. These methods cannot reliably distinguish among genes that have similar expression patterns but are under the control of different regulatory networks. Recently, the genome-wide location analysis of DNA-binding motifs offers new information for identifying regulatory relationships, such as *trans*-/*cis*- regulatory networks. The ChIP-chip method identifies the interactions between TFs and DNA binding regions, providing strong direct evidence for genetic regulation [10,11]. Although helpful, the usefulness of binding information is also limited, because the presence of the regulator at a promoter region indicates binding but not necessarily function. The environment conditions under which these TFs will interact with the *cis*-elements (DNA-binding motifs) are still not clear. The regulator may act positively, negatively or not at all [12].

Elucidating the regulation of genes and eventually deciphering the entire gene regulatory network will reveal the functions of genes during internal transcriptional processes and responses to external environmental stimuli. However, in order to analyze the functions of a target gene of interest, one first needs to understand the gene regulation network, which is a formidable task by conventional methods. The initial step towards the goal of understanding gene regulation is to identify the relationship between a TF and its target genes. Many TFs bind to specific sites in the genome to regulate gene expression. For example, they bind to specific motifs on promoter sequences and recruit chromatin modifying complexes and the transcription apparatus to initiate RNA synthesis [13-15]. The reprogramming of gene expression that occurs as cells move through the cell cycle, or when cells respond to changes in their environments, is effected in part by changes in the DNA binding status of *trans*-acting activators. Recently, the databases such as SCPD [16] and TRANSFAC [17] have been established to collect information from the literature about TFs with regard to their target genes and binding sites. However, experimental identification of TFs and their functions is slow and laborious. Therefore, prediction methods have become increasingly important, especially after the emergence of high throughput technologies, such as DNA microarrays, and binding site motif information [10,11]. Nguyen and D'haeseleer [18] have integrated genome-wide location data and motif binding sequence to infer the strength dependency of motif position and orientation and then focus on individual motif regulatory ability of the target gene. In this study, our goal is to develop an efficient systematic method that can integrate these data sources to detect TFs and their synergistic activities to gain more insight into the mechanism via constructing *trans*-regulatory networks of TFs responding to an environmental change.

Recently, Bussemaker *et al*. [19] proposed to use a linear regression model to identify binding motifs correlated with gene expression. Although this method successfully discovered some motifs corresponding to known binding sites and predicted some new motifs, they did not find their TF functions and interactions in diverse environments. The ability to adapt to osmotic changes in the surrounding medium and the heat shock due to sudden environmental temperature change is fundamental to life. To properly control gene expression, the cell has to sense osmotic or thermal changes and transmit the signal to the nucleus. Recently, TFs that are required for the stimulation of gene expression after the osmotic and heat shock have been described [20]. In recent studies, systems biology method and computational systems biology schemes have been widely used to construct dynamic models for gene regulatory networks [2-5,21-24]. In this study, utilizing the binding site motif information [10,11] and the microarray data [25,26] of different environmental stresses, we detect the activities of TFs under different stresses by quantifying their regulatory abilities and interactive activities. From the systems biology perspective, the TFs that are active under multiple environmental stresses can also be detected by a cross identification method. We can not only identify the individual interaction TF strength but also rank the TFs which are responsible to the specific stress. In addition, we can also estimate the crosstalk relationships among different pathways responsible to specific environmental stresses.

## Results

In this study, we consider seven different environmental or physiological conditions on yeast to demonstrate the performance of our method; the major conditions are osmotic stress, heat shock stress, hydrogen peroxide treatment, and cell cycle. TF activities are recognized by constructing an interactive dynamic model among the target genes and candidate TFs via a set of yeast DNA-binding motif information and microarray data. Because the temporal microarray data can be represented as a result of the interactive dynamic modeling, it is easy to discern the stress-specific TFs and the role of activator or repressor by estimating the regulatory abilities and interactive activities by the maximum likelihood estimation. The significant TFs responsible for a specific environmental stress is also detected for a target gene by minimizing the Akaike Information Criterion (AIC) to achieve the real order of the interactive dynamic model via the system identification method.

**Figure 1:**
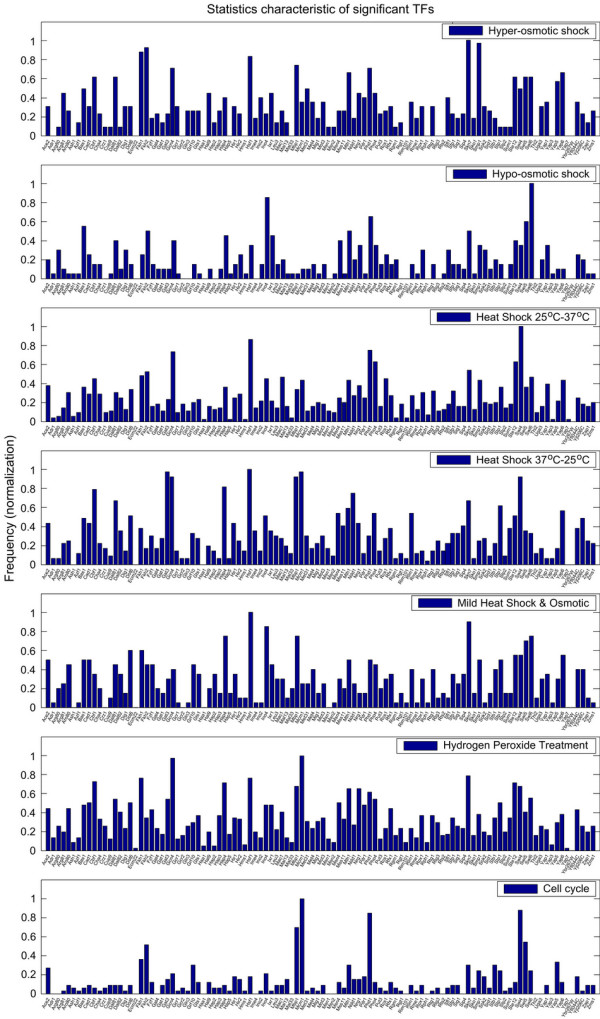
The statistics of frequencies of significant transcription factors (excluding the common transcriptional activators) are detected by the normalized frequencies of all target genes via our method. The results are from seven types of environmental or physiological stresses in *S. cerevisiae*.

**Figure 2:**
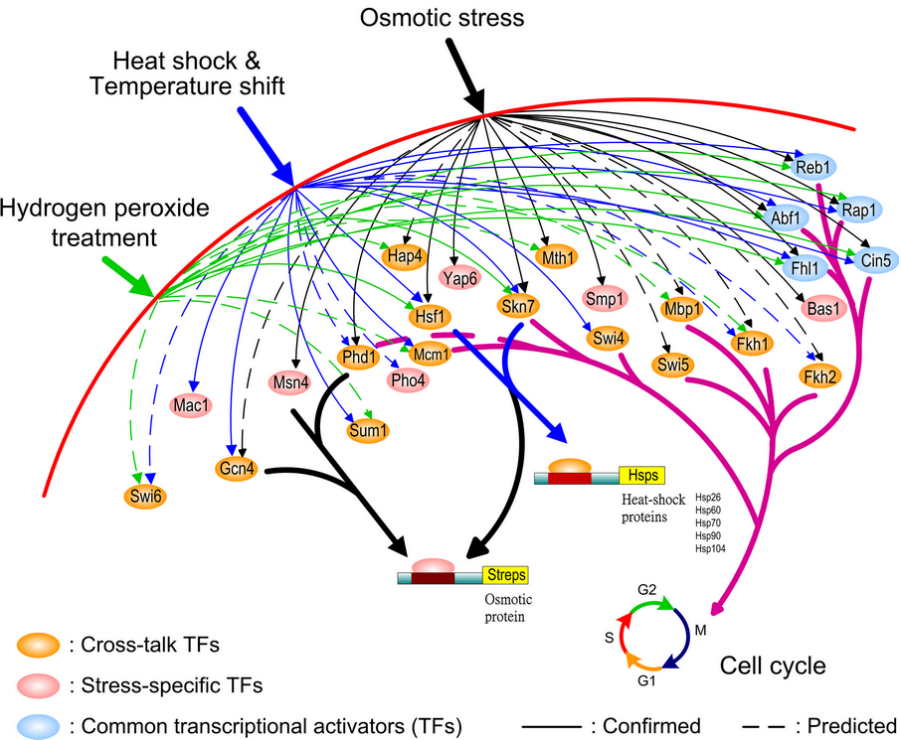
The interactions and cross-talks among significant TFs responding to different environmental stresses in the yeast. TFs in orange color have been shown to have cross-talks or interactions under different environment stresses; for example, Skn7 and Hsf1 are found to cooperate to achieve a significant induction of heat shock genes and hydrogen peroxide stress, respectively. Other TFs (pink color) play only the roles of controlling stress-specific genes, and the predicted TFs (dotted lines) are those genes which have not been experimentally confirmed yet.

In this work, the detected TFs are divided into two parts: (1) the stress-specific TFs, which are based on the statistical results in Figure 1, and (2) the common transcriptional activators in Table 1. Furthermore, the detected interactive activities among these TFs are presented in Table 2. In this study, we focus on detecting the stress-specific TFs and the common transcriptional activators that are always activative in the gene transcription process even in the absence of any specific stress; these common TFs can also be easily found by the conventional statistical method. For example, our proposed method can easily find the common TFs Abf1, Rap1, Cin5, Fhl1 and Reb1 [27-31] in osmotic shock, heat shock, hydrogen peroxide treatment and cell cycle in Table 1. The interactive activities of these TFs under different environmental conditions are ranked in interactive activities matrices in Table 2. In addition, our method also can order the relative roles of the TFs in stress-specific genes of the transcriptional regulatory system. In the following, we will analyze the stress-specific TFs in response to seven different stresses.

**Table 1:** The significant transcription factors in order by detected frequencies via our method under different environmental stresses in *S. cerevisiae*.

	**1**	**2**	**3**	**4**	**5**	**6**	**7**	**8**	**9**	**10**	**Common transcriptional activators**				
Hyper-osmotic Shock	Skn7*	Smp1*	Fkh2	Fkh1	Hsf1*	Mbp1*	Phd1*	Gcn4	Yap6*	Mth1*	Abf1*	Fhl1*	Cin5*	Rap1*	Reb1*
Hypo-osmotic Shock	Swi6	Ino4	Phd1*	Swi5	Bas1*	Skn7*	Mth1*	Fkh2	Ixr1	Hap4	Abf1*	Fhl1*	Cin5*	Rap1*	Reb1*
Heat Shock from 25°C to 37°C	Swi4	Hsf1*	Phd1*	Gcn4*	Sum1*	Pho4	Skn7*	Fkh2	Fkh1	Swi6	Abf1*	Fhl1*	Cin5*	Rap1*	Reb1*
Temperature Shift from 37°C to 25°C	Hsf1*	Mcm1*	Gat3	Stb1	Mbp1*	Gcn4*	Hap4	Cbf1	Ndd1*	Skn7*	Abf1*	Fhl1*	Cin5	Rap1*	Reb1*
Mild Heat Shock at Variable Osmolarity	Hsf1*	Skn7*	Swi6	Ino4	Mbp1*	Hap4	Swi5	Fkh1	Dot6	Swi4	Abf1*	Fhl1*	Cin5*	Rap1*	Reb1*
Hydrogen Peroxide Treatment	Mcm1	Gcn4	Skn7*	Hsf1*	Fkh1	Hap4	Cbf1	Sum1	Swi6	Mbp1	Abf1*	Fhl1*	Cin5*	Rap1*	Reb1
Cell cycle	Mcm1*	Swi4*	Phd1	Mbp1*	Swi5*	Fkh2*	Fkh1*	Yap5	Stb1	Skn7*	Abf1*	Fhl1*	Cin5*	Rap1*	Reb1*

* The transcription factors confirmed by the literature or experimental results.

Common transcription activators are TFs that always activate without any specific stress induction and are also ordered by their regulatory significance.

**Table 2:** The ranked cooperativities of transcription factors under different environmental conditions: The number *l *denotes the *l*-th significant cooperation among these transcription factors. In this table only cooperative activities of the cell cycle are ranked.

	**Cooperativity ranking matrix of cell cycle**									
**Cell Cycle**	Fkh2	Mbp1	Mcm1	Msn4	Ndd1	Pho2	Rap1	Swi4	Swi6	Yap5

Fkh2		**-**	**-**	**-**	2*	**-**	**-**	**-**	**-**	**-**
Mbp1			**7**	**-**	**-**	**-**	**-**	6*	4*	**-**
Mcm1				**-**	1*	**-**	**-**	**-**	**-**	**-**
Msn4					**-**	**-**	**9**	**-**	**-**	**-**
Ndd1						**-**	**-**	**5**	**-**	**-**
Pho2							**-**	**10**	**-**	**-**
Rap1								**-**	**-**	**8**
Swi4									3*	**-**
Swi6										**-**
Yap5										

* The interactivities confirmed by the literature or experimental results.

### Significant TFs under different environmental changes

#### Under osmotic stress

Our study indicates that the yeast genes that respond to hyper- and hypo-osmotic shock are regulated by the major TFs listed in Table 1. Skn7 has a high detected frequency under an osmotic stress, implying that Skn7 is a strong regulator for osmotic stress. Experimental evidence suggests that Skn7p is controlled by the Sln1p-Ypd1p osmosensing phosphorelay system and osmotic signals [32]. The function of Skn7p is opposite to the pathway responding to high osmolarity but is in parallel to a pathway responding to low osmolarity. The Sln1p-Ypd1p-Skn7p system is a genuine two-component (or phosphorelay) system mediating osmotic responses to a transcriptional regulator [32]. Skn7 appears to function as a TF, because it can bind to promoter elements [33] and can activate the transcription of reporter genes [34]. This ability to activate transcription is influenced by mutations at the phosphorylation site (D427) of the Skn7 receiver domain, and this finding leads to the prediction that a histidine protein kinase directs the phosphorylation of Skn7 [34,35]. However, the identity of this kinase remains to be confirmed.

Smp1 is the second most important TF in response to the hyper-osmotic stress (Table 1). Exposure of *S. cerevisiae *to strong extracellular osmolarity activates the stress-activated high-osmolarity glycerol (HOG) in mitogen-activated protein kinase (MAPK) pathway, which is essential for cell survival upon osmotic stress. Yeast cells respond to osmotic stress by inducing the expressions of a very large number of genes, in which TF Smp1 binds to Hog1, under the control of MAPK. Experimental results confirm that the relevant Hog1 phosphorylation sites in Smp1 have an obvious effect on stress-regulated gene expression [36]. Hence, Smp1 also plays an important role in osmotic-stress responses.

Similarly, Yap6, a member of the yeast activator protein (YAP) family, and Hsf1, Phd1, Mbp1, etc. also have indirect experimental evidence to confirm that they are related to osmotic stress [30,37]. Hence, in the osmotic-stress environmental stimulation, our proposed method is very efficient to detect significant TFs.

#### Under heat shock stress

The heat shock stress is another external environment stimulus in general. In Table 1, Hsf1 is found in front of other TFs, indicating that Hsf1 is the most significant TF in response to heat shock stress. This prediction is in agreement with the experimental evidence that the expression of the major heat shock proteins (Hsps), which have been classified according to their molecular mass as Hsp104, Hsp90, the Hsp70 family, Hsp60, Hsp26, and Hsp12 [38], is controlled by Hsf1, which binds to *cis*-acting heat shock control elements (HSE) present in the promoters of these genes [39].

Similarly, Skn7 has previously been shown to play a role in the induction of heat stress-responsive genes in yeast. Hsf1 and Skn7 share certain structural similarities, particularly in their DNA-binding domains and at the adjacent regions of coiled-coil structure, which are known to mediate protein-protein interactions [40].

In addition, we find another TF Mac1 in Table 1, whose N-terminal region is highly similar to the copper and DNA binding domains of ACE1 and AMT1. Loss-of-function mutants of MAC1 have a defect in the plasma membrane Cu(II) and the reductase activity Fe(III), which are slow growing, respiratory deficient, and hypersensitive to heat [41]. Moreover, we can also find that the significant TF Gcn4 is also considered as a stress-responsive TF [42]. Obviously, these significant stress TFs can be detected by our method.

#### Under mixed stresses

We also investigated TFs under other stress conditions, including mild heat shock at variable similarity and hydrogen peroxide treatment. Using our method, we can easily find a very significant TF Skn7. Further, in the hydrogen peroxide (H_2_O_2_) treatment, we can also find Hsf1. These two TFs are always ranked as the first or the second in Table 1. Cells must survive under challenges from the environment with regard to heat and hydrogen peroxide. Skn7 has previously been shown to play a role in the induction of stress-responsive genes in yeast, e.g., in the induction of the thioredoxin gene in response to hydrogen peroxide [40]. These two regulatory *trans*-activators, Skn7 and Hsf1, share certain structural similarities, particularly in their DNA-binding domains and in the presence of adjacent regions of the coiled-coil structure, which are known to mediate protein-protein interactions [40]. Furthermore, Raitt *et al *. [40] showed that Skn7 can bind to the same heat shock regulatory sequences as Hsf1, and that Skn7 and Hsf1 cooperate to achieve a maximal induction of heat shock genes in response specific to oxidative stress and interact with each other in the nucleus under normal growth conditions as well as during oxidative stress.

#### Under cell cycle

Our method identified several TFs that have previously been identified as major cell cycle controlling TFs, including Mcm1, Swi4, Mbp1, Swi5, Fkh1, and Fkh2 [43-47]. These TFs are activators in the cell cycle. Besides, we also find the common transcriptional activators Abf1, Rap1 and Reb1, which also play important roles in the transcription of genes in the cell cycle of yeast [1]. For example, Abf1 has a positive regulation of the genes that are involved in protein synthesis and transport, glycolysis, fermentation, energy pathways and cell wall organization [16,48,49]. This Abf1 function was also inferred by Wei and Kaznessis [50]. On the other hand, Reb1 is an essential transcription factor that interacts with the CLB2 upstream regulatory sequence (URS) outside the G2/M control region [29]. Obviously, these cell cycle controlling TFs can be detected by our method.

### Synergistic TF pairs under specific environmental changes

We now consider the interactivities of these significant TFs in the cell cycle (Table 2). The interactivity between Mcm1 and Ndd1 has the strongest regulatory ability in the cell cycle. According to experimental data, Mcm1, together with Fkh2, recruits Ndd1 in late G2 and controls the transcription of G2/M genes [1,47]. The interaction between Fkh2 and Ndd1 has the second strongest regulatory ability according to our results. Furthermore, we also find strong interactivities between Swi4 and Swi6, and between Mbp1 and Swi6 (Table 2). According to the conventional results in the yeast cell cycle, complexes of Swi4 and Swi6 (SBF) as well as Mbp1 and Swi6 (MBF), both of which are heterodimers, are active during the G1/S phase [47,51]. Thus, the interactivity of TFs can be accurately detected by our method.

### Cross-talks and interactions

From the results in Table 1, we can find that many TFs are detected in different environmental conditions, including Skn7, Hsf1, Phd1, Hap4, Gcn4, Mbp1, etc. (Figure 2). It implies that they may be cross-talks or interactions among the stress response pathways, such as the high osmolarity glycerol (HOG) pathway and the heat shock response pathway. Although they respond to different conditions, they may be induced by the same TFs. This cross-talk phenomenon may imply interactions among TFs. In Table 1, we find that Skn7 and Hsf1 are detected in the heat shock stress and hydrogen peroxide treatment; a previous study [33] showed that Skn7 and Hsf1 cooperate to achieve the maximal induction of heat shock genes in response to hydrogen peroxide stress specifically. In addition, Skn7 and Hsf1 share certain structural similarities in their DNA-binding. Therefore, from Tables 1 and 2, we can infer that several TFs may have cross-talks and interactions in different pathways induced by environmental stresses (Figure 2).

## Discussion

In contrast to current methods, our new method is capable of extracting significant regulatory abilities and interactivities of TFs under different environmental conditions. For this reason, the analysis and interpretation of output expression profiles become straightforward, so that our method has a high potential for application. It may also be useful for studying the cross-talks between pathways controlled by the same regulatory TFs.

The contributions of this study include the followings: (1) a nonlinear dynamical model is developed for a transcription regulatory system in terms of regulatory abilities and interactivities among TFs, (2) a systematic identification method is proposed to detect the specific-stress regulation TFs and their interactivities, (3) a searching method for TFs is developed by the proposed dynamic transcriptional regulatory system, (4) the proposed method can rank the frequencies of TFs that correspond to a specific stress and thus can identify the major TFs. Similarly, it can rank the frequencies of interactions between TFs and thus identify the major TF pairs, and (5) cross-talks and interactions among different environment-stress inducing pathways can also be estimated by detecting the regulatory TFs. The main advantage of our method over current methods is that the transcriptional regulatory system is constructed with the genome-wide structure using the expression profiles and ChIP-chip data, and the gene regulatory system can obtain extra dynamic information to meet the dynamic regulations and interactivities of genetic networks under environmental stresses.

However, we have weaknesses or poor results in some cases. For example, in Table 1, we find TFs Ixr1 and Dot6. These detected specific-stress TFs cannot be confirmed by current experimental data to validate the function annotation. A possible reason may be that the use of both cubic spline interpolation to avoid overfitting and linear transformation of microarray data in our scheme has introduced new noises and distortions. Furthermore, some estimated TFs and their interactivities may influence the specific-stress pathway indirectly but cannot be detected by experiment directly. In addition, the binding motif information might be incomplete, and the TF-binding site number and location are not sufficient to construct the complete transcriptional regulatory system with regard to the genes under study. Therefore, the lack of complete TF binding site information makes it difficult construct an accurate dynamic interactive equation to estimate TF activities. The estimated false positive and false negative rates of these first 10 TFs for specific environmental stresses are given in Table 3. Since the TFs of the cell cycle and hyper-osmotic shock are better known at present, the estimates of their TFs can be evaluated. From Table 3, we find a small false positive rate and a larger false negative rate. The performance may be improved by finding more TFs in the literature or/and by increasing the estimate of significant TFs. On the other hand, the current literature and experimental data are not sufficient to confirm our results by the proposed method. At present, we only provide the prediction results of the TFs' interaction for future research.

**Table 3:** The confirmed TFs, false positives and false negatives of the estimated 10 significant TFs in the cell cycle and the hyper-osmotic stress.

Conditions	Confirmed number	False positive number	False negative Number	Reference from Saccharomyces Genome Database (SGD), Lee *et al*. [11] and Gat-Viks *et al*. [57].
cell cycle	8 TFs	2 TFs	4 TFs	12 TFs.
hyper-osmotic	7 TFs	3 TFs	9 TFs	16 TFs.

In the future, if more complete binding motif data and more accurate and longer gene expression profile data become available, we will be able to improve the construction of the transcription regulatory system and get more interactive information of TFs under different environmental conditions. Also, our approach may be extended to constructing transcriptional regulatory systems in more diverse conditions and more complex eukaryotes. After transcription factors and their interactivities are accurately detected by explicit dynamical equations, some applications will become straightforward.

## Conclusions

In this study, a dynamic system model is developed to describe the regulatory ability and interactivity of TFs for each gene expressed in a specific stress due to environmental changes. Based on the proposed dynamic system model and microarray data as well as information of all possible binding sites, we could find significant stress-specific and cell cycle-controlled TFs through ranking the frequencies of TFs of all genes, which are expressed under a specific stress. Similarly, the significant interactivities of TFs under a specific stress are also found by ranking the frequencies of interactions of all TFs. Most of the results are confirmed by the literature. Further, the cross-talks of TFs among different stresses are also detected, which deserve further research. The proposed TF detection method is a systems biology approach because all possible TFs of all genes are considered through microarray and ChIP-chip data and a system identification method is used to estimate the parameters of the dynamic system model. The results of our proposed approach are suitable for deciphering regulatory functions, interactivities and cross-talks of TFs that respond to different environmental stresses.

## Methods

The dynamics of a *cis*-regulatory circuit of target genes can be modeled by a differential equation, which is well established and analyzed [52-55]. The TFs' responses to a specific environmental or physiological change are detected by our method through the modeling of the *trans/cis *regulatory network of target gene expressions. The approach is divided into two steps. The first step is to find the gene expression profiles of microarray data [25,26] for the genes that respond to the specific environmental change. The main DNA binding site information was compiled from the data set of Lee *et al*. [11] with a P-value ≤ 0.0015. In these ChIP-chip data, for a P-value of 0.001 the frequency of the false positives is 6% to 10%, but the frequency of false negatives is 33%. Combining with binding site motif information, we can construct dynamic equations of the stress-perturbed transcriptional regulatory system. In order to find the TFs that respond to a specific environmental stress, we need to prune the transcription regulatory system by fitting the dynamic equations with microarray data. In the second step, the coefficients of the dynamic equations can be identified by the maximum likelihood estimation (MLE) algorithm to represent the regulatory abilities and interactivities of TFs of the stress-induced target genes. Based on the AIC order detection, TFs with significant coefficients in the dynamic equation of a target gene are considered significant TFs of the target gene. However, the significant TFs responding to a specific environmental stress need to include all significant TFs of all target genes responding to the specific stress. In this situation, the statistics of the frequencies of significant TFs are necessary for all target genes that respond to the specific environmental stress. Therefore, we calculate the statistical frequencies of the significant coefficients which are identified from all target genes responding to a specific environmental stress or physiological change. Then the TFs with high frequencies of significant coefficients of all target genes represent the significant TFs to the specific environmental stress. The detail is given in the following section.

### Dynamic modeling of a transcriptional regulatory system

The dynamic model of the transcription regulatory system of target gene *i *under a specific environmental stress is modeled by the following interactive dynamic equation

$$Y^{i}(t+1)=\sum_{p=1}^{v}\alpha_{p}^{i}\cdot X_{p}(t)+\sum_{p=1}^{v-1}\sum_{q=p+1}^{v}\beta_{p,q}^{i}\cdot X_{p,q}(t)-\lambda^{i}\cdot Y^{i}(t)+\varepsilon^{i}(t)$$

where *Y*^*i *^(*t*) represents the mRNA expression level of target gene *i *at time point *t*,. *X*_*p *_(*t*), *p *∈ {1, 2,..., *v*}, represents the input regulation functions of *v *candidate TFs binding to the target gene, i.e., the *v *TFs that can bind to the binding sites of target gene *i *via ChIP-chip data are considered as the candidate TFs of target gene *i *in the dynamic Equation (1). $\alpha_{p}^{i}$ indicates the possible regulatory ability or kinetic activity of the *p*-th TF in target gene *i*. *X*_*p, q *_(*t*) is the possible regulatory function of cooperative TFs, and is described by the following nonlinear equation of interactivities between TFs *p *and *q*

$$X_{p,q}(t)=:\sqrt{X_{p}(t)\cdot X_{q}(t)}$$

and $\beta_{p,q}^{i}$ denotes the regulatory ability (or kinetic activity) of the cooperative TFs *p *and *q*. The parameter *λ*^*i *^indicates the degrading effect of the present state value *Y*^*i *^(*t*) on the next state value *Y*^*i *^(*t *+ 1), and *ε*^*i *^(*t*) denotes a stochastic noise owing to model uncertainty and fluctuation of mRNA microarray data in the target gene. In this study, we assume *ε*^*i *^(*t*) is a Gaussian white noise with zero mean and unknown variance $\sigma_{i}^{2}$.

The *v *TF-DNA interactions in Equation (1) are based on the main binding sites (P-value ≤ 0.0015) of ChIP-chip data of the target gene. Since CHIP-chip data only indicates the promoters to which the TFs putatively bind, the *v *TFs in the dynamic Equation (1) are only the candidate TFs of gene *i *. These *v *candidate TF-DNA interactions should be detected by mRNA microarray data via the AIC model order detection through the system identification method below. Only the significant TF-DNA interactions are considered in the dynamic equation after system identification and the insignificant TF-DNA interactions will be deleted from the dynamic model. Then the refined dynamic model will represent the TF regulations in gene expressions under the specific environmental stress.

**Figure 3:**
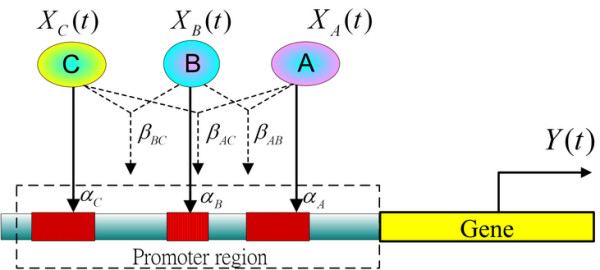
Dynamic model of the transcriptional regulatory system of a target gene. The candidate regulatory TFs of the target gene were obtained from the genome-wide TF binding location data by chromatin immunoprecipitation [10,11]. A binding transcription factor *p *has a regulatory ability *α*_*p *_and interactivities with TF *q *to produce the regulatory ability *β*_*p*, *q*_. The microarray data for these TFs provide the inputs *X*_*p *_(*t*) of the transcriptional regulatory system and produce the output *Y *(*t*) of the target gene.

For example, the transcription regulatory system of target gene *i *under a specific environmental stress is illustrated in Figure 3, including the possible regulatory functions and interactivities of its TFs. The *trans/cis *regulatory system in Figure 3 is modeled by the following interactive dynamic equation

*Y *(*t *+1) = *α*_*A *_*X*_*A *_(*t*) + *α*_*B *_*X*_*B *_(*t*) + *α*_*C *_*X*_*C *_(*t*) + *β*_*A, B *_*X*_*A, B *_(*t*) + *β *_*A, C *_*X*_*A, C *_(*t*) + *β*_*B, C *_*X*_*B, C *_(*t*) - *λY *(*t*) + *ε *(*t*)

*X*_*A *_(*t*), *X*_*B *_(*t*) and *X*_*C *_(*t*) are the incident regulations of TFs *A*, *B*, *C*, respectively. Interactivities *X*_*A, B *_(*t*), *X*_*A, C *_(*t*) and *X*_*B, C *_(*t*) denote the interactivities among TFs A, B and C.

$$\begin{array}{c} X_{A,B}(t)=:\sqrt{X_{A}(t)\cdot X_{B}(t)}\\ X_{A,C}(t)=:\sqrt{X_{A}(t)\cdot X_{C}(t)}\\ X_{B,C}(t)=:\sqrt{X_{B}(t)\cdot X_{C}(t)} \end{array}$$

The biological meaning of Equation (1) is that the next mRNA expression level of the target gene is due to the result of the productions of the present transcriptional regulatory functions *α*_*A *_*X*_*A *_(*t*) + *α*_*B *_*X*_*B *_(*t*) + *α*_*C *_*X*_*C *_(*t*) + *β*_*A, B *_*X*_*A, B *_(*t*) + *β*_*A, C *_*X*_*A, C *_(*t*) + *β*_*B, C *_*X*_*B, C *_(*t*).

### Remark 1

The possible combinations or cooperation of 3 TFs or more in dynamic Equation (1) will increase the difficulty in the parameter estimation process, especially, in the case of a large number of TFs. Therefore, they are not included directly in our dynamic model. However, if the cooperation of 3 TFs exists, it will be expressed by the following three cooperative 2 TFs simultaneously, i.e., *X*_*A, B *_(*t*), *X*_*A, C *_(*t*) and *X*_*B, C *_(*t*) in the case of Equation (3).

According to Lee *et al*. [11], there are 106 possible TF candidates to be considered in Equation (1). For the transcriptional regulatory systems in Equation (1), because of multiple regulatory inputs *X*_*p *_(*t*) and a large number of interactivities *X*_*p, q *_(*t*), one can estimate the regulatory abilities of the corresponding transcription factors by constructing the complete system dynamic model for each target gene with environmental and physiological changes. By integrating the dynamic equations of time-series transcriptional regulatory systems for *n *time points *t *= 1, 2, …, *n *in response to certain environment condition (stress), we obtain the following array dynamic matrix form for target gene *i*

Y_*i *_= Φ·Θ_*i *_+ E

where

$$\begin{array}{c} {\text{Y}}_{i}=\left[\begin{array}{c} Y^{i}(1)\\ Y^{i}(2)\\ \vdots \\ Y^{i}(n-1)\\ Y^{i}(n) \end{array}\right],\Theta_{i}=\left[\begin{array}{c} \alpha_{1}^{i}\\ \alpha_{2}^{i}\\ \vdots \\ \alpha_{v}^{i}\\ \beta_{1,2}^{i}\\ \vdots \\ \beta_{v-1,v}^{i}\\ \lambda^{i} \end{array}\right],\text{E}=\left[\begin{array}{c} \varepsilon^{i}(0)\\ \varepsilon^{i}(1)\\ \vdots \\ \varepsilon^{i}(n-2)\\ \varepsilon^{i}(n-1) \end{array}\right]\\ and\;\Phi \left[\begin{array}{cccccc} X_{1}(0) & X_{2}(0) & \cdots & X_{1,2}(0) & \cdots & -Y^{i}(0)\\ X_{1}(1) & X_{2}(1) & \cdots & X_{1,2}(1) & \cdots & -Y^{i}(1)\\ \vdots & \vdots & & \vdots & & \vdots \\ X_{1}(n-2) & X_{2}(n-2) & \cdots & X_{1,2}(n-2) & \cdots & -Y^{i}(n-2)\\ X_{1}(n-1) & X_{2}(n-1) & \cdots & X_{1,2}(n-1) & \cdots & -Y^{i}(n-1) \end{array}\right] \end{array}$$

Y and Φ can be obtained from the microarray data of gene *i *and the other possible genes under a specific environmental stress. Equation (1) can then be used to estimate the transcription factors' regulatory abilities and interactivities Θ_*i *_and the noise variance $\sigma_{i}^{2}$ for target gene *i *by the following *Maximum Likelihood Estimation Algorithm *[56]:

$${\bar{\Theta }}_{i}={\left(\Phi^{T}\Phi \right)}^{-1}\Phi {\text{Y}}_{i}$$

$${\bar{\sigma }}_{i}^{2}=\frac{1}{n-1}{\left({\text{Y}}_{i}-\Phi {\bar{\Theta }}_{i}\right)}^{T}({\text{Y}}_{i}-\Phi {\bar{\Theta }}_{i})$$

### Remark 2

Although the maximum likelihood estimation method can help us quantify the regulatory abilities of all the possible regulatory TFs on target genes, we still don't know exactly how significant an estimated regulator can be regarded as a true regulator. In order to achieve the goal for determining whether a regulator is significant or not, a statistical approach based on model validation is proposed for evaluating the significance of our model parameters to prune the rough *v *TFs identified from ChIP-chip data. In this study, a statistical approach, namely the Akaike Information Criterion (AIC), is employed to validate the model order (or the number of TFs) to determine the significant TFs of our dynamic equation in (1).

The Akaike Information Criterion (AIC), which attempts to include both the estimated residual variance ${\bar{\sigma }}_{i}^{2}$ and model complexity *v *in one statistics, decreases as the residual variance ${\bar{\sigma }}_{i}^{2}$ decreases and increases as the number *v *of TFs increases. As the expected residual variance decreases with increasing *v *for nonadequate model complexities, there should be a minimum AIC around the correct number *v *of TFs. For a dynamic model with *v *parameters to fit with data from *n *data samples, the AIC can be written as follows [56]

$$\text{AIC}(v)=\log\frac{1}{n-1}{\left({\text{Y}}_{i}-\Phi {\bar{\Theta }}_{i}\right)}^{T}({\text{Y}}_{i}-\Phi {\bar{\Theta }}_{i})+\frac{2v}{n}$$

After the statistical selection of *v *= *s*_*i *_TFs minimizing the AIC in (8) (i.e., *v *= *s*_*i *_achieves the minimization in (8)), we can choose these *s*_*i *_TFs as significant TFs for target gene *i *and the remainders are false positives.

In order to avoid the overfitting in the parameter estimation in Equation (1), the cubic spline method is employed in this study to interpolate data points *n *which should be much larger than the number of parameters to be estimated. After $\bar{\Theta }$ is estimated from Equation (6), the regulatory abilities and interactivities can be identified for the corresponding transcriptional regulatory system under a specific environmental or physiological change. Then, one chooses the largest *s *parameters $\bar{\alpha_{k}^{i}}$ of the significant regulatory abilities in absolute value with regard to the *i*-th target gene, in the following order

$$\left|\bar{\alpha_{1}^{i}}\right|\geq \left|\bar{\alpha_{k}^{i}}\right|\geq \cdots \geq \left|\bar{\alpha_{s_{i}}^{i}}\right|$$

Then the corresponding significant transcription factors for target gene *i *are given by the set

$${\left[\begin{array}{cccc} tf_{1}^{i} & tf_{k}^{i} & \cdots & tf_{s_{i}}^{i} \end{array}\right]}_{1\times s_{i}}$$

where the first element represents the TF that has the most significant regulation to the target gene expression under the specific environmental or physiological condition. If there are *W *target genes in response to the specific environmental or physiological changes, we can get a set of significant TFs for this specific condition by counting the frequencies of $tf_{k}^{i}$ which appear in the set in Equation (10) for *W *target genes responding to the same environmental condition, i.e., the distribution of significant TFs of *W *genes is given by the following frequency matrix

$${\left[\begin{array}{ccccccccc} tf_{1}^{1} & tf_{2}^{1} & 0 & tf_{4}^{1} & \cdots & \cdots & tf_{k}^{1} & \cdots & tf_{v}^{1}\\ 0 & tf_{2}^{2} & tf_{3}^{2} & 0 & \cdots & \cdots & 0 & \cdots & 0\\ tf_{1}^{3} & 0 & tf_{3}^{3} & tf_{4}^{3} & \cdots & \cdots & 0 & \cdots & tf_{v}^{3}\\ \vdots & & & \vdots & & & \vdots & & \vdots \\ tf_{1}^{W} & 0 & 0 & tf_{4}^{W} & \cdots & \cdots & tf_{k}^{W} & \cdots & tf_{v}^{W} \end{array}\right]}_{W\times v}$$

**Figure 4:**
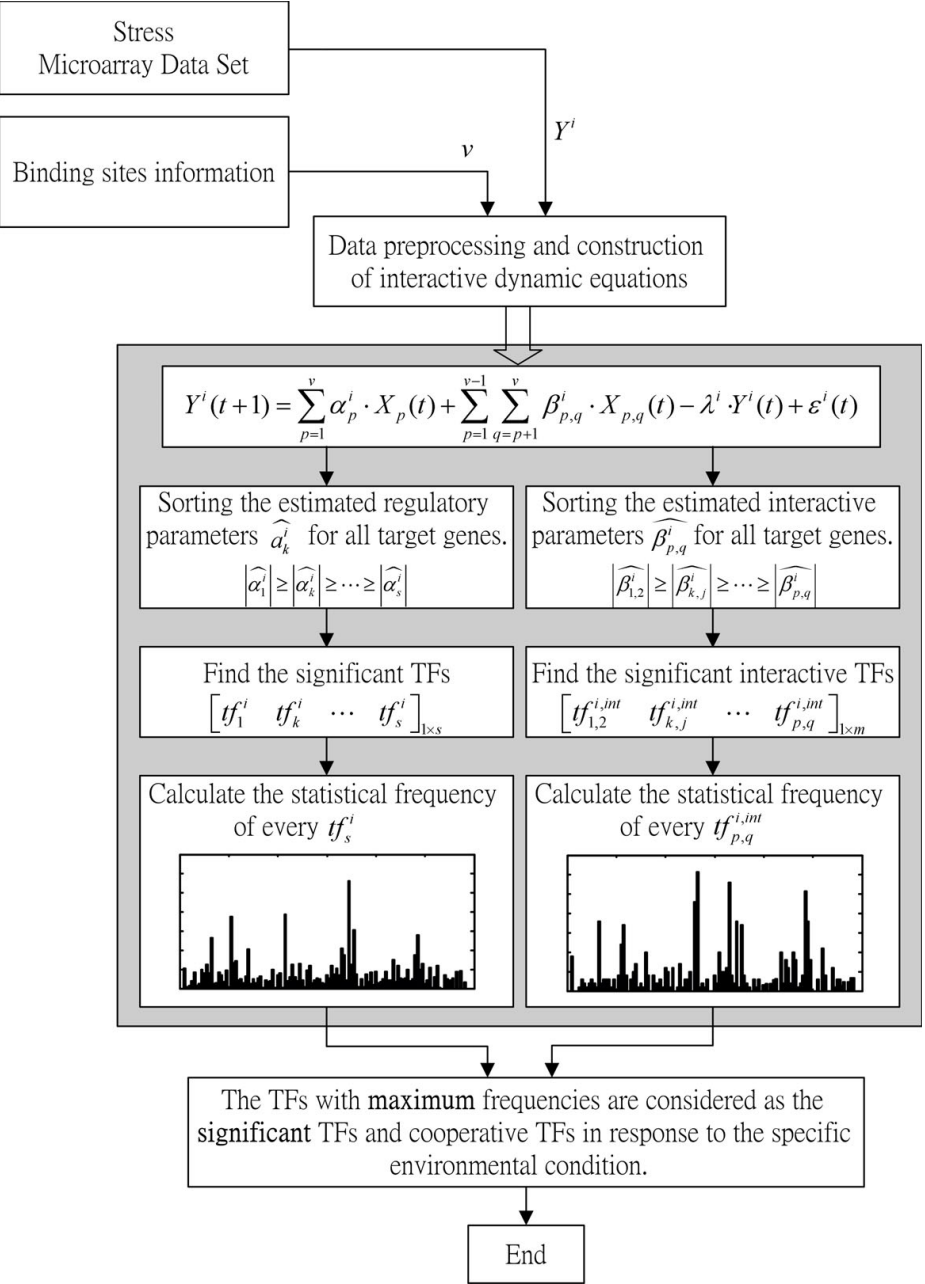
The overall flowchart of the modeling, identification and finding the significant TFs of a dynamic transcriptional regulatory system.

The *i*-th row in the above matrix denotes the distribution of *s*_*i *_significant TFs of the *i*-th target gene that is expressed under the specific environmental conditions. We count the numbers in each column to find the frequencies of their corresponding TFs to *W *target genes in response to this environmental condition. The normalization of the frequencies of TFs is shown in Figure 1 for seven types of environmental or physiological stresses in *S. cerevisiae*. In the results, the first *s *TFs with maximum frequencies in each column of Equation (11) are considered as the most significant transcription factors in response to the specific environmental condition. The significance of each TF is according to the frequency of appearance in each column of the frequency matrix in Equation (11). In this study, for the convenience of table listing, only 15 significant transcription factors are listed for a specific environmental or physiological condition, i.e., *s *= 15. In other words, we can detect the significant TFs active at different environment conditions. The flowchart for modeling, statistics of frequency and significant TFs finding is shown in Figure 4.

For the same reason, in order to detect interactivities of cooperative TFs under a specific environmental or physiological change, the interactivities $\beta_{p,q}^{i}$ between TFs *p *and *q *of the *i*-th target gene in Equation (1) among the *s*_*i *_significant TFs are described in order as follows

$$\left|\bar{\beta_{1,2}^{i}}\right|\geq \left|\bar{\beta_{k,j}^{i}}\right|\geq \cdots \geq \left|\bar{\beta_{p,q}^{i}}\right|$$

where $\bar{\beta_{p,q}^{i}}$ denotes the estimate of interactivity from Equation (6) between TFs *p *and *q *in the promoter region of target gene *i *under a specific environmental condition. Then the corresponding significant interactivities among cooperative TFs of target gene *i *are given by the set

$${\left[\begin{array}{cccc} tf_{1,2}^{i,int} & tf_{k,j}^{i,int} & \cdots & tf_{p,q}^{i,int} \end{array}\right]}_{1\times m_{i}}$$

where the superscript *int *denotes the interactivity of TFs *k *and *j*, and the first $tf_{k,j}^{i,int}$ has the most interactive regulation contribution to the target gene expression under the specific environmental or physiological condition.

Suppose only the *m *significant interactivities among cooperative TFs are chosen for this specific environmental or physiological condition. Then the interactivity matrix for the cooperative TFs of *W *target genes responsible to a specific environmental stress is given as follows

$$\left[\begin{array}{ccccccccc} tf_{1,2}^{1,int} & tf_{1,3}^{1,int} & 0 & tf_{1,5}^{1,int} & \cdots & \cdots & tf_{k,j}^{1,int} & \cdots & tf_{(v-1),v}^{1,int}\\ 0 & tf_{1,3}^{2,int} & tf_{1,4}^{2,int} & 0 & \cdots & \cdots & 0 & \cdots & 0\\ tf_{1,2}^{3,int} & 0 & tf_{1,4}^{3,int} & tf_{1,5}^{3,int} & \cdots & \cdots & 0 & \cdots & tf_{(v-1),v}^{3,int}\\ \vdots & & & \vdots & & & \vdots & & \vdots \\ tf_{1,2}^{W,int} & 0 & 0 & tf_{1,5}^{W,int} & \cdots & \cdots & tf_{k,j}^{W,int} & \cdots & tf_{(v-1),v}^{Z,int} \end{array}\right]$$

Each row in the above matrix denotes the distribution of *W *significant interactivities among TFs of one gene that is expressed under the specific environmental or physiological condition. In the results, the first *m *significant interactivities among cooperative TFs with maximum frequencies in each column of Equation (14) are considered as the significant cooperative TFs in response to the specific environmental or physiological condition. The significance of each interaction among cooperative TFs is according to the frequency of appearance in each column of the matrix in Equation (14). In the interactivity case, for convenience, we choose only 10 significant cooperations among TFs that are listed for a specific environmental condition, i.e., *m *= 10 (see Table 2 in the cell cycle case). In this study, for the convenience of table listing, we choose *s *= 15 and *m *= 10 in Equation (11) and Equation (14), respectively.

From the systematic analysis above, we can detect *s *significant transcription factors and *m *significant cooperative TF pairs from microarray data for yeast under different environmental stresses. From these significant transcription factors and their significant cooperation, we can construct different stress-induced pathways and cross talks in Figure 2 to gain much insight into protective mechanisms of yeast under different environmental and physiological changes.

## Authors' contributions

LHL carried out the model design and computation of this study, and drafted the manuscript. HCL participated in the design of the study and drafted the manuscript. WHL amended and improved the design and the presentation of the study. BSC gave the topic and suggestions and was responsible for the entire study. All authors read and approved the final manuscript.
